# Long term surgical outcome for persistent pupillary membranes with associated ocular abnormalities: a retrospective case series study

**DOI:** 10.1186/s12886-021-01990-8

**Published:** 2021-05-25

**Authors:** Yu-Te Huang, Hui-Ju Lin

**Affiliations:** Department of Ophthalmology, China Medical University Hospital, China Medical University, 2 Yuh-Der Road, Taichung City, 40447 Taiwan

**Keywords:** Persistent pupillary membranes, Surgical technique, Visual rehabilitation

## Abstract

**Background:**

Deprivation amblyopia is a great concern in hyperplastic persistent pupillary membranes (PPM) which blocked visual axis. Other ocular abnormality may accompany and further hinder the visual development of the infants.

We evaluate the long-term visual prognosis and complications in patients with dense PPM and other associated abnormalities treated with early surgical intervention and timely visual rehabilitation.

**Methods:**

Medical records of patients with surgical removal of PPM from 2000 to 2020 and also receiving visual rehabilitation were retrospectively reviewed. Besides visual axis blocked PPM, patients combined with other amblyopic risk factors or ocular abnormalities were included.

Due to preparation for subsequent lens extraction if an underlying cataract was present, the surgical settings including the instruments and wound direction were similar to cataract surgery. All patients were enrolled in a visual rehabilitation program as soon as possible. The results including sex, age, timing of operation, initial and final visual acuity, refractive errors, and complications were recorded.

**Results:**

Seven cases of five patients were included in this case series. Mean age at surgery was 42.3 ± 21.1 months (range, 5 to 66 months) and the post-operative follow-up period was 4.9 years (range, 1.2 to 8.2 years).

The patient age at time of surgery ranged from 2.5 months to 2.5 years (mean, 14 months). Mean postoperative follow-up was 5.3 years (range, 2.5–8 years). There were no intra-operative and post-operative complications. Final BCVA varied with a mean value of 0.29 logMAR (range, 0 to 1 logMAR). An associated ocular abnormality of ametropia and strabismus led to the best visual prognosis.

**Conclusions:**

In patients with PPM, there were no significant complications in any patient using our technique. The surgical settings are easier to handle and more familiar with pediatric surgeons. Besides deprivation with patching, early PPM intervention and timely visual rehabilitation achieve the best visual prognosis in patients associated with risk of ametropic and strabismic amblyopia.

**Trial registration:**

This retrospective, interventional case series study was conducted at China Medical University Hospital between April 1, 2000 and April 31, 2020. (IRB number: CMUH109-REC2–069).

## Background

Persistent pupillary membranes (PPM) are the most commonly seen congenital abnormality of the iris in newborns. The prevalence rate is high, with 95% of newborns being affected. However, it rarely has any visual development significance because regression is usually completed before the first year of life [1]. Fine diaphanous remnants at the pupillary margin are a common finding in older children and adults and they have no clinical significance.

In cases with thick, heavily pigmented, centrally located PPM, intervention is always considered to clear the visual axis and reduce the risk of deprivation amblyopia. Pharmacologic therapy with mydiasis and YAG laser treatments are all available options with relatively good outcomes [2]. Surgery is the last resort due to the risks of general anesthesia, intraoperative bleeding, iatrogenic cataract formation and post operation endopthamitis [3–5].

PPMs are mostly isolated but association with other congenital abnormalities has still been reported, including congenital cataract, high refractive errors, glaucoma, micropthalmos and iris coloboma [6, 7]. In such circumstances, surgical success cannot guarantee a good visual outcome.

To achieve the ideal final visual outcome in such complicated cases, pediatric visual rehabilitation may be another additional viable option. In this case-series study, we report the long-term surgical outcome combining a visual rehabilitation program in children with PPM and other ocular abnormalities.

## Methods

### Ethical approval

This retrospective, interventional case series study was conducted at China Medical University Hospital (CMUH) between April 1, 2000 and April 31, 2020. The study was performed in accordance with the World Medical Association’s Declaration of Helsinki and the study design was approved by the Institutional Review Board of China Medical University Hospital (IRB number: CMUH109-REC2–069). Owing to the retrospective design of the study and the use of deidentified patient information, the review board waived the need for written informed consent.

### Study population

Patients less than 4 years of age that underwent surgical intervention of the thick PPM covering the visual axis were retrospectively reviewed. The inclusion criteria were surgically treated PPM combined with other amblyopic risk factors or ocular abnormalities. For example, the refractive status was considered amblyogenic according to American Academy of Ophthalmology guidelines. Simple PPM cases were not included in this study.

The main exclusion criteria were (1) lost to follow up (2) previous interventions including YAG-laser treatment.

### Study procedure

A detailed full ophthalmologic exam was performed at the initial visit, including anterior segment evaluation, intraocular pressure, and dilated fundus exam. Age, sex, associated ocular abnormality, pre-operation refraction status and visual acuity were recorded. The Snellen chart was the main tool for visual acuity assessment, but ocular behaviors (ability of fixation and following objects) were applied to younger patients.

The severity and location of the PPM were evaluated and described by one experienced examiner (H.J, Lin). Pre-operative treatment including (1) Tropicamide 1% (Mydriacyl; Alcon, Belgium) were applied four times a day. (2) Patching of unilateral or bilateral asymmetric lesions were based on the patient’s condition. Patents were re-evaluated within 2 months. Surgical indications were as follows: poor retinoscopic reflex, decreased visual acuity, visual axis blockage or opening less than 1.5 mm.

### Surgical technique

Due to possible underlying cataract and anterior lens capsule damage during surgery, all cases underwent preparation for cataract surgery. If a cataract presented during the operation, lens extraction could then be carried out.

All procedures were performed under general anesthesia. Starting from instillation of miotics, a 1.5-mm clear cornea incision was then made. Viscoelastic agent was injected into the anterior chamber to create a working space beneath the pupillary strands to lift off PPM. Then, a 3-plane 2.2 mm limbus wound was made as the main wound. The adhering strands were carefully peeled from the anterior lens capsule with intraocular scissors. Then, strands at the pupillary margin were cut with the same equipment. Pupillary strands were removed by Kelman-McPherson forceps. The viscoelastic agent was washed out and the cornea was sutured with 10–0 Nylon.

Post operation medication included topical levofloxacin (Cravit; Santen, Japan), prednisolone acetate 1% (Econopred Plus; Alcon, Belgium) four times day and tropicamide 1% (Mydriacyl; Alcon, Belgium) once a day for 1 month.

### Visual rehabilitation programs

General conditions were managed as usual, including the timing of patching or prescribing glasses. Visual rehabilitation programs began as soon as the patient was suitable (mostly the training program starting around the age of 4). A CAM vision stimulator was given once a week at 30 min a time. Vectograms were also given once a week at 30 min a time. All the vision therapy continued until the visual acuity was stable.

### Assessment of clinical outcome

Post operation examination was arranged at 1 day, 4 days, 2 weeks, and 1 month after surgery. Refraction status, visual acuity and IOP were recorded if possible. Anterior segment photographs were obtained afterwards. Then patients were scheduled for outpatient department at intervals of 3 to 6 months.

## Results

### Demographic and clinical characteristics of patients

A total of 5 patients with 7 eyes were included. These cases were suggested to have PPM with a high risk of amblyopia. There were three males (60%) and two females (40%) with a mean age of 33.9 months present in our department (range, 3 to 45 months). Table 1 summarizes the key characteristics and visual outcomes of the individual patient. Mean age at the time of surgery was 42.3 months (range, 5 to 66 months). Associated ocular abnormality included both eyes inferior oblique overaction (IOOA), exotropia and severe astigmatism (− 7.25D in the right eye and − 6.25D in the left eye) in patient 1. Patient 2 was complicated by severe astigmatism (− 2.75D in the left eye) and Patient 3 had right eye keratoconus. Patient 4 had large optic nerve coloboma in both eyes and Patient 5 had severe anisometropia (5D). The visual acuity before surgery successfully obtained in four eyes of two patients was 0.54 logMAR. The remaining three patients were too young to assess viable visual acuity.

**Table 1:** Summary of the key characteristics and visual outcomes of the patients

Case	OD/S	Age of Diagnosis (Ms)	Age of Surgery (Ms)	PreOP Refraction	PreOP Axial length (mm)		PreOP VA (LogMAR)
1	OD	45	47	−1.25-2.5 × 10	20.20		0.52
2	OS	45	47	−1.5-3.75 × 175	20.70		0.7
3	OS	37	38	+ 2.5–0.75 × 150	19.86		< 1^+^
4	OD	3	5	−5.25-0.75 × 115	N/A		< 1.3*
5	OD	42	64	−5.25-1.25 × 140	22.53		0.52
6	OS	42	66	−7.50-1.25 × 20	23.01		0.4
7	OS	23	29	+ 3.75–0.50 × 145	20.55		< 1^+^
Case	Associated ocular condition			Final Refraction	Final VA (LogMAR)	Follow up (Ys)	Other Tx
1	XT + IOOA & high astigmatism			−1.50-7.25 × 180	0	5.8	Strabismus correction
2				−4.00-6.25 × 175	0	5.8	
3	High astigmatism			+ 2.50–2.75 × 155	0	5.9	No
4	Keratoconus			+ 0.00–0.75 × 30	0.22	8.2	Scleral lens
5	Optic nerve coloboma & Congenital nystagmus			−5.25-3.25 × 160	0.52	3.8	No
6				−6.00-3.75 × 175	1	3.7	
7	High anisometropia			+ 1.50–1.50 × 180	0.3	1.2	No

N/A: Not available.

* By OKN drums as screening test with VA > 20/400

+ By CSM methods as screening test: CSUM (Central, steady, unmaintained) as VA > 20/200

*OD* right eye, *OS* left eye, *VA* visual acuity, *XT* exotropia, *IOOA* inferior oblique overaction, *logMAR* logarithm of the minimum angle of resolution

### Postoperative clinical status during follow-up

After a mean follow-up period of 4.9 (range, 1.2 to 8.2) years, the best-corrected visual acuity (BCVA) at the final visit was 0.29 logMAR, and the mean refractive error was − 5.46 diopters. Table 2 demonstrated the demographic and clinical characteristics of all patients. All patients successfully cleared the visual axis and removed all the iris strands. No intraoperative complications were observed. Slight bleeding presented when gently cutting the iris strand but this stopped spontaneously. No residual hyphema, iatrogenic cataract or infection were observed.

**Table 2:** Demographic and clinical characteristics of all patients

Characteristics	Value (*n* = 7)
Male: female	3:2
Age at diagnosis (Ms)	33.9 ± 15.6 *
Age at surgery (Ms)	42.3 ± 21.1 *
Intraoperative complications	0*
Postoperative complications	0*
Postoperative follow-up period (Ys)	4.9 ± 2.2 *
Preoperative axial length (mm)	21.14
Initial Visual acuity (LogMAR)	0.54 ± 0.10 ^+^
Final Visual acuity (LogMAR)	0.29 ± 0.37
Initial spherical equivalent (D)	−2.84 ± 4.30
Final spherical equivalent (D)	−3.46 ± 4.00

*Bilateral eyes were operated; the value was counted twice

+ Only 4 cases could obstain viable data

*logMAR* logarithm of the minimum angle of resolution, *D* diopters

Values are presented as number or mean ± standard deviation

**Fig. 1:**
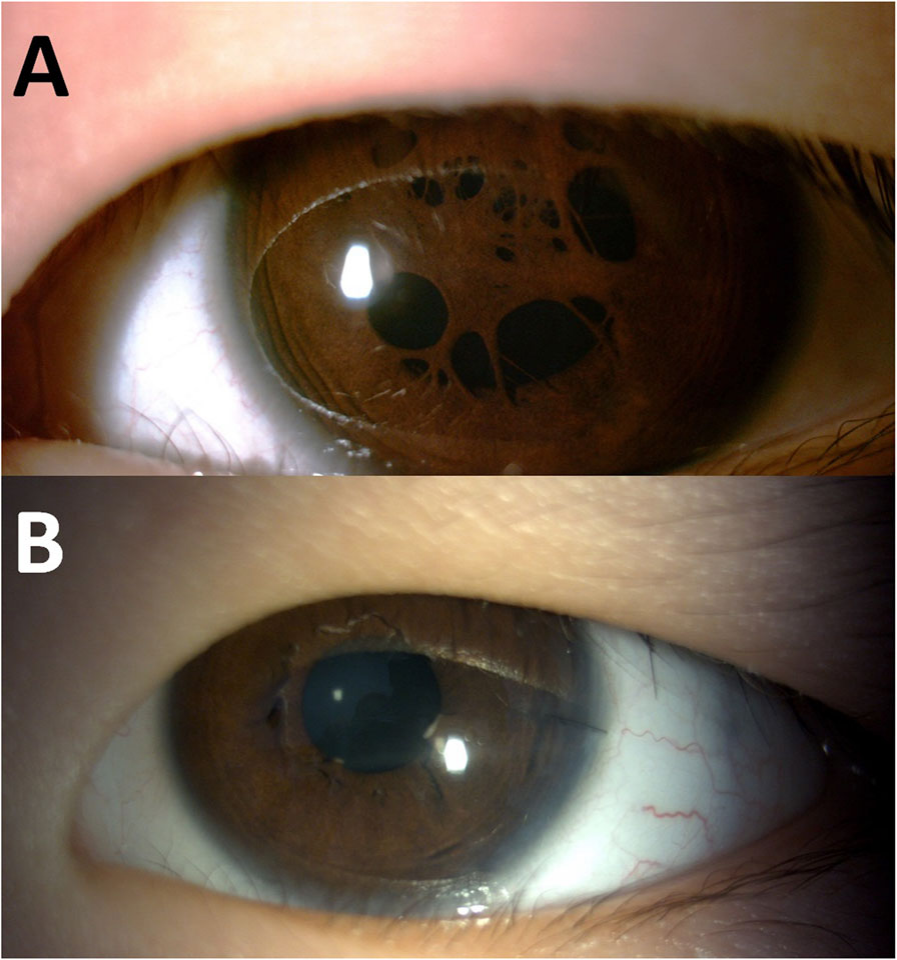
Case 7. **A**: Before operation; **B**: One month after operation

**Fig. 2:**
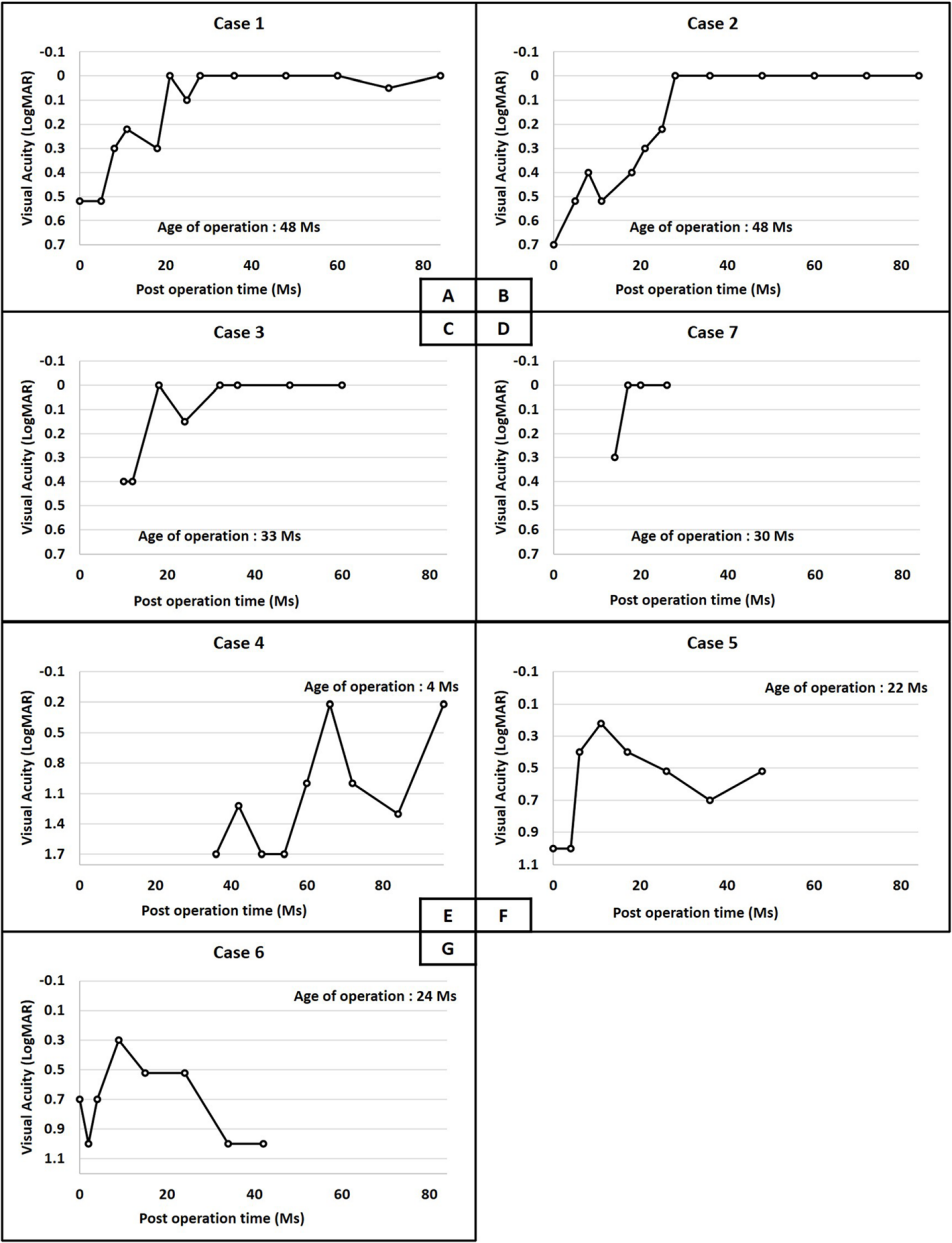
Subsequent post-operative VA during the follow-up period. Figure 2**A** to 2**D** represents the cases with better final visual outcome (Case 1–3,7). Figure 2**E** to 2**G** represents the cases with worse final visual outcome (Case 4–6)

Patient 1 (Case 1 and 2) showed the best outcome in this series. She presented at the age of 4 with dense PPM complicated by exotropia and IOOA. An operation was first performed with PPM then correction of strabismus in the following years. A visual rehabilitation program was initiated soon after PPM removal. After following up for nearly 6 years, her BCVA improved to 0.00 logMAR despite residual high astigmatism (− 7.25D in the right eye and − 6.25D in the left eye) (Fig. 1). Patient 2 (Case 3) was similar to Patient 1, except for it being less severe upon unilateral presentation. A final BCVA of 0.00 logMAR was achieved by successful operation, visual rehabilitation program and patching. Patient 3 presented to us at the age of 3 months. Since the PPM was so dense that it clearly obscured the visual axis, an operation was arranged at the age of 5. Keratoconus became apparent several years later with a follow-up period of 8 years. He is still amblyopic with BCVA of 0.22 logMAR (on sclera lens). Patient 4 (Case 5 and 6) had the poorest outcome in this series. This was a case with bilateral congenital nystagmus and large optic nerve coloboma. PPM was less severe, so conservative treatment was arranged. Her BCVA failed to improve at 0.52 logMAR in the right eye and 0.4 logMAR in the left eye. After discussing with her parents, the operation was carried out with initiation of a visual rehabilitation program. However, after following up for nearly 4 years, visual outcome was less effective with 0.52 logMAR in the right eye and 1 logMAR in the left eye. Patient 5 (Case7) was a case with high ametropia (4D). Even with a lower follow up duration, he showed promising early results in terms of visual development (Fig. 2).

## Discussion

The pathogenesis of PPM is still a mystery. Some theories suggest a transitory membrane, composed of strands from major circulus arteriosus of the iris with the mesenchyme failing to regress at the end of eight and a half months of gestation age [8]. This membrane, which replaces the vascular tunica, lies between the corneal endothelium and lens epithelium. The remnants between the iris collarette form PPM [9]. The diagnosis is quite straight forward clinically, however, some rare congenital conditions should be considered as a differential diagnosis. For example, pupillary-iris-lens membrane with goniodysgenesis and congenital idiopathic microcoria all present with excessive iris tissue but do not extend from the iris collarette [10, 11]^.^

Treatment options include conservative treatment with or without mydriatics. Pharmacological mydriasis, occlusion therapy and close observation all played some role in selected cases [2, 6, 12]. Another non-surgical option is YAG-laser [13]. Besides the risk of hyphema and pigments dispersion, it may not be efficacious in thicker PPM. Most importantly, it is reserved for older patients and mainly for cosmetic reasons. Therefore, it is not practical in these cases of visual development. Previous studies considered a pupillary diameter of 1.5 mm necessary for visual development and refrained from amblyopic [12]. So, in our practice, the indications for an operation mostly followed this rule.

Surgical techniques and equipment varied according to the surgeon’s preferences. Incisions ranged from a single 4 mm to 1.5 mm wound, to a pair of small incisions [9, 14–16]. We suggested “cataract-extraction- like” incision wounds, which is one 2.2 mm main wound with another smaller side port incision [17]. The advantage is pediatric surgeons are familiar with this intraocular operation setting because it is the same as in cataract surgery. Further, much more working space can be achieved compared with a single entry. The most important advantage compared with single port entry is if an underlying cataract is discovered during the operation, sequential cataract operations can be done without changing wound settings. As for separating the PPM from the iris, vitrectors, vitrectomy scissors, and intraocular scissors are all recommended instruments [14–16]. We prefer intraocular scissors for two main reasons. First, nonautomated equipment reduces the risk of iatrogenic complications, as supported by previous research [14]. Second, the cost is much lower compared with vitrectors and non-retina specialists may be unfamiliar with vitrectors and vitrectomy scissors.

In our study, the associated ocular abnormalities differed from previous cohorts. To our knowledge, no other study has reported congenital nystagmus, optic nerve coloboma, IOOA and keratoconus associated with dense, thick PPM. Previous studies reported latent nystagmus, esotropia exotropia and hydrocephalus, Pallister-Killian syndrome and nasolacrimal duct obstruction [14, 15]. Further studies may determine whether a curtain genetic effect or association may contribute to these conditions.

Lens opacity following the operation is the main concern because removing iris strands from the anterior lens capsule might inevitably cause some minor trauma, which contributes to this complication. However, our cases had no induced lens opacities over a relatively long period of follow-up time. Other studies also reported comparable results. Courtney et.al reported 10 eyes with no cataract occurrence after a mean postoperative follow-up of 5.3 (range, 2.5 to 8) years [14]. Lee et.al showed 32 eyes with only 6.3% lens opacity rates in a mean follow-up of 6.5 (range, 4.0 to 14.8) years [15]. One hypothesis came from a histopathological finding reported by Ramappa et al. The theory suggested the lens capsule was special in some cases, and the surface of the iris strands adhering to the lens can have identical histologic features from the lens epithelium itself [9]. It also explained removing a firmly attached PPM from the lens capsule with an intact lens epithelium.

Visual rehabilitation comprises all kinds of interventions, focusing on improvement of visual abilities, visual development, and coping with visual disabilities [18]. The best result comes with a multidisciplinary approach, involving physicians, optometrists, occupational therapists and most important of all, the patients. The program can be either done at home or trained in the hospital by a teacher. In this case series, all patients were introduced to curtain training programs (CAM vision stimulator and Vectograms) as soon as possible. As expected, the best outcome (Final BCVA of 0.00 logMAR) was found in patients with only refractive errors as associated ocular abnormalities (Case 1 and 2). Even in the case with very high astigmatism (>7D), the outcome was excellent. However, in highly myopic (> 6 D) cases (Case 5 and 6), the outcome could be less favorable with final BCVA all < 0.18 logMAR in our and other reports [6]. Strabismic amblyopia can also be avoided through timely surgical correction (Case 1 ) [19]. The same results are also supported by a previous study [6]. In cases with ocular abnormalities other than refractive status, the outcomes are more variable. PPM is considered less important in causing amblyopia if treated promptly and properly. Cases 5 and 6 were the clearest examples that amblyopia was caused by large optic disc coloboma and congenital nystagmus. Timing PPM removal and a visual rehabilitation program were fruitless in this case.

According to the population-based normative data from previous studies, the normative visual acuities of 12 m, 18 m, 24 m, 36 m, 48 m, 60 m were 0.57, 0.51, 0.48, 0.24, 0.1, 0 respectively [20]. Considered the case with better final VA (Case 1–3, 7), the visual acuities were all lagging behind the age norm VA (Pre-OP VA / Age norm VA): Case 1: 0.52/ 0.1, Case 2: 0.7/ 0.1 Case 3: < 1 / 0.24 Case 7: < 1 / 0.4. After the operation, it took another 21, 28, 18 and 17 weeks respectively to catch up to the age norm VA. Due to the lack of a control group, we could not fully eliminate the possibility of age as a confounding factor. On the other hand, the cases with poor final VA (Case 4–6) never caught up to the age norm VA due to underlying diseases.

The primary limitation of this study is its retrospective nature. Therefore, initial VAs were not completed, especially in non-verbal infants. However, the OKN drum and CSM methods were carried out as a screening tool. Those with detailed and regular followed up cases were included and clear and detailed data were available for analysis. The second limitation is the lack of a control group. Due to the concern of deprivation amblyopia with delayed treatment, a more proactive indication was made. Reynolds et.al suggested prompt therapeutic intervention for PPM, even within the first several months [21].

The strength of this paper is its long-term follow up period and focus on how associated abnormalities influence visual outcomes. There were no significant complications in any patient using our technique. The surgical setting and equipment are much easier to handle and more familiar to pediatric surgeons.

## Conclusions

In patients with PPM, there were no significant complications in any patient using our technique. The surgical settings are easier to handle and more familiar to pediatric surgeons. Besides patching, early PPM intervention and timely visual rehabilitation achieve the best visual prognosis in patients associated with risks of ametropic and strabismic amblyopia. We suggest early intervention and timely visual rehabilitation to achieve the best vision prognosis in patients with significant persistent pupillary membrane.

## Data Availability

The datasets used and/or analyzed during the current study are available from the corresponding author on reasonable request.

## Abbreviations

PPM: Persistent pupillary membranes
IOOA: Inferior oblique overaction
BCVA: Best-corrected visual acuity
